# “Out of the frying pan and into the fire” – the UKFPO’s recent changes are a short-sighted response to a complicated problem

**DOI:** 10.1186/s12909-024-05594-w

**Published:** 2024-06-20

**Authors:** Oliver Skan, Kiran Saini, Edward Armstrong

**Affiliations:** 1 London North West University Healthcare NHS Trust, London, UK; 2 Oxford University Hospitals NHS Foundation Trust, Oxford, England

**Keywords:** Medical school examinations, Foundation program applications

## Abstract

In June 2023, the UK Foundation Programme Office announced that the previous method of ranking students based on their educational performance measure and situational judgement test performance would be superseded by a preferencing algorithm that disregards academic merit. We outline our strong objections to this policy.

In June, the UK Foundation Programme Office (UKFPO) announced major changes to the way that medical students will be awarded places in the two-year foundation programme. Previously, students were ranked according to their educational performance measure (EPM), a surrogate marker of their academic performance during university, combined with the results of their situational judgement test (SJT), a 140-minute test taken nationally in December. Under the new system, students will simply rank their preferred foundation programmes, and an algorithm will then give as many students as possible their top choice – known as ‘preference informed allocation’ (PIA) [1].

We posit the ideal foundation matching process would be a valid and reliable assessment of a student’s ability, predict future success in the foundation programme and beyond, and have a positive educational impact. It is difficult to assess the utility of the EPM + SJT in predicting success during the foundation programme, not least due to the paucity of data expressing how ‘well’ a foundation doctor performs during their placements. However, by analysing what data is routinely collected, it has been found that the SJT + EPM has at least some predictive validity – both a candidate’s EPM and SJT score are associated with increased odds of passing foundation training during the Annual Review of Competency Progression [2]. Indeed, a recent meta-analysis has found that evidence supports the use of SJTs in medical selection [3].

There are clear arguments against the EPM + SJT model. Having been through finals and the SJT as students ourselves only three years ago, we can attest to just how stressful this period was. Flaws in the SJT are well documented [4]​. Questions are notoriously rigid and subjective, with similar questions often marked differently based on small changes to wording, with even subject matter ‘experts’ often struggling to agree on the correct responses [5]. While designed to not require revision, most would agree that preparation is possible, although the nature of the examination makes this a difficult and frustrating process that often coincides with medical school finals.

Data suggesting that white candidates score significantly higher than minority candidates are also concerning [6]​, although should be taken in the context of data suggesting that there is a systemic problem with differential attainment amongst ethnic minority students across all medical assessments [7]. This is a complex issue that will require systematic approaches to change, but does not appear to be unique to the SJT.

During the exam a student could find their hard-earned decile ranking fall dramatically, undoing five or six years of hard work at medical school. This led many to decry the SJT as unmeritocratic and unfairly reflective of a candidate’s ability.

Using the EPM as a tool for determining foundation placements also has its flaws. Students are ranked only within their medical school, which can foster unhealthy competition between students at a time when we should be promoting the sort of collaborative working expected in the NHS. It also lacks standardisation across universities despite medical schools having different entry standards and post-graduate outcomes [8].

However, despite the flaws in the SJT + EPM model, moving to a completely unmeritocratic system is a short-sighted response to a complicated problem.

A central pillar of the UKFPO’s argument is that their modelling suggests “a higher number of applicants (79.47%) will obtain their first choice Foundation School when compared to EPM + SJT score-based allocation (73.90%)” [9]. This ignores the changes in student behaviour that will result from the new system. Previously students were first assigned a deanery, and then allocated rotations based on their ranking within that deanery. This incentivised lower-ranking applicants to apply to less competitive deaneries, as ranking higher within that deanery would give them more agency over their rotations, as opposed to being a lower-ranked applicant within a more competitive deanery. While ‘tactical ranking’ is artificially disguising some candidates’ true preferences for deaneries, it serves a useful purpose to help the system cope with excessive demand for some deaneries.

Without this ‘tactical ranking’ behaviour, applications to popular deaneries will surely rise. Current UKFPO modelling suggests only around 5% more students would get their top preference with PIA, giving little leeway to buffer these changes to preferencing behaviour. It is also important to note that when you look at the percentage of students getting any one of their top three deaneries, the UKFPO’s own modelling shows PIA actually performs worse than EPM + SJT (89.63% vs. 90.35%) [10]​​.

Concerns about students’ mental health and the levels of stress during exams are valid and must be acted on. Calls for a more compassionate medical school experience that prioritises students’ mental health above their academic ranking are welcome [11]​. There is certainly a balance between a level of stress compromising mental health and a lack of motivation preventing students from reaching their full potential. It is not being cynical to suggest that for some students, knowing you only need to pass would result in them taking their ‘foot off the gas’, as the incentives to excel are severely diminished. This is a sentiment shared on medical school social media forums [12]​.

Medical school finals should be (and are) competency based – they demand that candidates reach a certain level at which they can safely start in the medical workforce. The EPM, while flawed, provides an incentive for medical students to work beyond the minimum required to pass. On an individual level, exceeding these passing requirements may make the transition to foundation years easier. On a wider systems level, we believe that encouraging excellence amongst medical students is an important step in creating the clinical leaders, clinician-scientists, and medical innovators of tomorrow.

Medical school also serves as a ‘safe’ environment for students to learn to manage their time and resources effectively, building resilience in preparation for the realistic longer-term challenges of medical practice. These challenges include difficult post-graduate examinations, often attempted whilst working full time, where the emphasis on candidates’ mental health is much reduced. Attempting to reduce competitive academic demands during medical school, therefore, may make some students less well-prepared for the long-term rigors of the profession.

Moreover, no other bottleneck of medical selection is treated like this. It is illogical to declare candidates unable to handle the pressures of medical school exams while simultaneously being fit to compete for core training, registrar training numbers, consultant posts, and indeed to get into medical school in the first place. Instead of universally removing all academic ranking, resources should be targeted specifically at those who are struggling, to help guide them through medical school. Removing the pressure of competing against your immediate peers would also greatly reduce examination stress at medical school.

Instead, PIA will remove all agency from medical students, who are now totally stripped of their ability to influence where they will live and work. This total loss of agency causes a different, but very real, form of stress for students to contend with. The UKFPO have swapped a flawed system that required refinement, for a lottery.

Another of the UKFPO’s justifications is that their survey findings indicate that most respondents prefer PIA to the current system (66.40% vs. 33.60%) [9]. This does not necessarily indicate that PIA is a good option – only that students hope it might be better than the flawed SJT + EPM model. The question is, is there an alternative?

Could a national standardised finals examination be the answer? The introduction of the UKMLA from 2024 to 2025 has already demonstrated there is capacity to integrate novel national exams into medical school finals. Students would be motivated to work hard while removing the crushing pressure of competing against your immediate peers. This score could then be supplemented by non-standardised assessments throughout medical school to ensure that one final examination is not overly weighted. In an ideal world, these assessments would also include more ‘non-technical’ attributes such as communication skills and ability to work in a team – somewhat ironically, it is SJTs that were intended to test these skills.

We welcome the statement from the UKFPO that the new system will be under constant review, and changes can be made if needed. The UKFPO’s modelling should be made available, as well as modelling that accounts for potential changes to preferencing behaviours, which we believe is likely. If the absence of ‘tactical ranking’ ultimately means that fewer students are happy with their deanery, it would be interesting to see if students would still choose PIA over EPM + SJT – we feel they would not.

## Data Availability

All data is in the public domain and the sources of this data are referenced in the article.
